# Proteomic Analysis Comparing Effect of Feeding Practices on the Milk Fat Globule Membrane Proteins from *Camelus dromedarius*

**DOI:** 10.3390/foods15030506

**Published:** 2026-02-01

**Authors:** Afshan Masood, Ibrahim O. Alanazi, Assim A. Alfadda, Salini Scaria Joy, Fabio Mazzotti, Ousman Mahmood Ousman, Hicham Benabdelkamel

**Affiliations:** 1 Proteomics Resource Unit, Obesity Research Center, College of Medicine, King Saud University, P.O. Box 2925, Riyadh 11461, Saudi Arabia; afsmasood@ksu.edu.sa (A.M.); aalfadda@ksu.edu.sa (A.A.A.);; 2 Healthy Aging Research Institute, Health Sector, King Abdulaziz City for Science and Technology, Riyadh 11442, Saudi Arabia; ialenazi@kacst.edu.sa; 3 Department of Medicine, College of Medicine, King Saud University, P.O. Box 2925, Riyadh 11461, Saudi Arabia; 4 Strategic Centre for Diabetes Research, College of Medicine, King Saud University, Riyadh 11461, Saudi Arabia; sjoy@ksu.edu.sa; 5 Dipartimento di Chimica e Tecnologie Chimiche, Università Della Calabria, Via P. Bucci Cubo 12/C, I-87036 Arcavacata di Rende, CS, Italy; fabio.mazzotti@unical.it

**Keywords:** milk fat globular membrane (MFGM), proteome, *Camelus dromedarius*, feeding practices, MALDI-TOF mass spectrometry

## Abstract

Feeding systems are key factors shaping the biochemical and bioactive composition of camel milk, yet their impact on the milk fat globule membrane (MFGM) remains insufficiently understood. This study employed 2D-DIGE MALDI mass spectrometry-based proteomics to compare the MFGM protein profiles of milk obtained from desert-fed camels (DFCs) (*n* = 5) and farm-fed camels (FFCs) (*n* = 5), of the Waddah breed. The proteomic analysis revealed statistically significant changes in abundance (ANOVA *p* ≤ 0.05; fold change ≥ 1.5) in 40 proteins between the two groups. Nine proteins were upregulated and thirty-one proteins were downregulated in the DFCs compared to the FFCs. The DFC group showed a statistically significant increase in proteins including erythropoietin, keratin isoforms, COX16, and αS2-casein, while levels of Lactotransferrin, Lactadherin, Toll-like receptor 2, and superoxide dismutase among others were decreased. Increased abundance of proteins seen in the MFGM component of the DFC group is probably associated with stress adaptation related to desert feeding. Our findings from this pilot study provide proof-of-concept that the composition of proteins in the MFGM fraction varies according to feeding environment and practices. Farm feeding improved the bioactive protein content, whereas DFC milk contained higher levels of proteins related to stress adaptation. These insights have important implications and should be further evaluated in larger cohorts.

## 1. Introduction

Human health relies on a healthy diet and good nutrition, which are crucial for providing the energy and nutrients necessary for the body and brain to function, supporting growth and development, and protecting against diseases like heart disease, diabetes, and cancer. Proper nutrition strengthens the immune system, helps maintain a healthy weight, and improves mood. A balanced diet incorporates milk and dairy products among its dietary components [1]. Milk is a nutrient-dense food containing macro- and micronutrients comprising carbohydrates, fats, proteins, minerals, water, nutrients, and catalysts. The high nutritional quality of milk proteins is based on the presence of important amino acids, as well as their high digestibility and bioavailability. In general, across the globe, 81% of the milk production comes from cows while another 20% is obtained from different species including sheep, ass, horse, yak, goat, bison, and camel [2].

Among the various dairy animals, camel milk has been recently gaining popularity because of its high nutritional value and therapeutic effects and numerous nutraceutical benefits. Previous research has documented that camel milk is considered the best in terms of the nutritional value for human consumption and accounts for 0.4% of the global milk that is consumed. In the Middle East, the most common camel breed is the one-humped Camelus dromedarius, followed by the two-humped Camelus bactrianus, both of which are integral to communities in arid regions such as the Middle East and the Arabian Peninsula [3,4,5]. In comparison to bovine milk, camel milk is known to be lower in fat content, with a higher proportion of unsaturated and long-chain fatty acids, and a favorable mineral composition (e.g., higher iron, zinc) that may contribute to improved bioavailability. Beyond its nutritive contribution, camel milk is traditionally known to have therapeutic properties. This is particularly attributed to its chemical composition, particularly to its protein, peptide, fatty acid, high concentration of immunoglobulins, serum albumin, lactoferrin, and insulin-like peptides, as well as antioxidant compounds [6,7,8]. Antioxidants in camel’s milk have been reported to prevent symptoms of metabolic syndrome and even may prevent cardiovascular disease [9,10]. Previous studies also suggest that it has antimicrobial, antiviral, antioxidant, anti-inflammatory, antidiabetic, and anticancer effects and is hypoallergenic compared to bovine milk as it lacks β-lactoglobulin [11].

The quality of the milk, its quantity, nutrient profile, yield, and therapeutic properties largely depends on a number of factors including the genetic breed, stage of lactation, environment, and the diet or feeding methods and practices [12,13,14,15,16,17,18]. The diversity in feeding methods, especially for camels, not only shapes milk yield but also its nutritional composition, health benefits, and suitability for different populations and uses. Seasonal fluctuations and feed composition also impact the bioactive compounds, fatty acid profiles, and overall health benefits of camel milk. Camels, particularly valued in arid and semi-arid regions, have two primary feeding systems, desert-fed or free-browsing and indoor/domesticated farmed camels. Camel milk composition comprising 87% water, 3.5% fat, 3.4% protein, 4.4% lactose, and 0.78% ash demonstrates an evolutionary adaptation to arid environments through the concentration of essential solids and minerals [19].

Traditionally, desert-fed camels (DFCs) graze freely across vast, harsh landscapes, browsing on native shrubs, salty pastures, and hardy desert grasses, and predominantly consuming rough, fibrous native plants. Their adaptive foraging behavior enables them to consume a wide variety of plants, feeding from higher ground levels and often moving from one type to another and surviving extended dry periods with minimal water intake [20,21]. This feeding behavior influences the fatty acid profiles, mineral content, and even some bioactive compounds in their milk. In contrast, indoor-fed camels are maintained on formulated diets that can include alfalfa pellets, fruits, vegetables, Bermuda hay, grains, carrots, apples, dates, dried grass, wheat, oats and other grains, salt blocks, and vitamin and mineral supplements [22]. These controlled feeding systems typically result in higher milk yields and more consistent macronutrient composition, particularly with respect to protein and fat content, and tend to have a more stable and elevated nutritional composition, with increased protein and fat content. These indoor regimes aim to boost productivity, reduce nutritional deficiencies caused by seasonal scarcity, and elevate reproductive success [22]. Variability in milk composition across feeding methods is common in other species and has been observed in cattle, buffalo, and other dairy ruminants. Studies show that cows fed with concentrates and high-quality forage (zero grazing or component feeding) produce milk with higher protein and fat content compared to cows on natural pasture alone. However, the amplitude of these differences is generally more pronounced in camels due to their adaptation to desert environments, which fundamentally alters their metabolism and milk characteristics in response to heat stress, hydration, and nutritive fluctuations [22,23].

Milk fat globule membrane (MFGM) is a complex three-layered membrane surrounding the milk fat globule membrane, which originates from the luminal surface of mammary epithelial cells contains 1–4% of total milk proteins, phospholipids, triacylglycerol, enzymes, and micronutrients [24]. It plays a critical role in maintaining the physical stability of milk fat and acts as a rich source of biologically active molecules. Because the MFGM is directly derived from the mammary cell membrane, its protein composition reflects the physiological, nutritional, and environmental status of the animal. Evidence has been presented that MFGM has a beneficial effect on intestinal health, improving skin condition, and combats hypercholesterolemia, obesity, and inflammation [25]. As MFGM is a reservoir of membrane proteins, it is considered as a potential target for identifying signaling and secretory pathways associated with mammary glands [26]. In recent years, camel milk has been the subject of extensive research due to its potential nutraceutical and medical benefits. Therefore, investigating how different feeding systems such as desert grazing or DFC versus intensive indoor feeding affect the MFGM proteins provides vital insight into how management and nutrition shape the nutritional quality and functional value of camel milk.

Proteomic analysis has emerged as a powerful approach for exploring the complex protein composition of milk and its fractions, particularly the MFGM. Mass spectrometry-based proteomics enables comprehensive identification and quantification of these proteins, revealing subtle differences linked to species, lactation stage, diet, and environmental stress. In camel milk research, proteomics is especially valuable, as the camel MFGM remains less characterized than its bovine or caprine counterparts [27,28,29]. Through comparative proteomic profiling, it is possible to detect feeding-system-related changes in proteins associated with antioxidant activity, metabolism, and immune function, which in turn influence the nutritional and biofunctional quality of the milk. We previously compared differences in the MFGM proteome between different breeds of *Camelus dromedarius*, namely Waddah and Safra [30]. However, systematic evaluation of the effects of feeding environments and management practices on the camel MFGM proteome remains limited, particularly within the Saudi Arabian context

Therefore, the present study was designed to address this knowledge gap. Given the functional importance of the milk fat globule membrane, we aimed to characterize and compare the proteome of the MFGM component of camel milk obtained from *Camelus dromedaries* (Waddah breed) raised under two distinct feeding systems: desert-fed (free-browsing) and indoor farm-fed conditions in Saudi Arabia.

## 2. Materials and Methods

### 2.1. Camel Milk Samples Collection

Camel milk (250 mL) was sampled from 10 healthy *Camelus dromedarius* species of the Waddah breed with different feeding practices (desert-fed camels (DFCs) (*n* = 5), farm-fed camels (FFCs) (*n* = 5)). Sampling was conducted during morning sessions (06:00 to 08:00 am) to capitalize on the higher daily yield characteristic of morning milk. Strict hygienic protocols were observed; the teats were cleaned, disinfected with 70% alcohol, and dried prior to collection. The initial milk squirts were discarded to minimize bacterial contamination from the teat canal. Samples were obtained in sterile 0.5 L containers and transferred immediately to the laboratory via a standard icebox. To prevent biological degradation and microbial growth, the specimens were treated with 0.1% sodium azide and agitated gently. Subsequently, the samples were fractionated into 50 mL labeled Falcon tubes and frozen at −20 °C (Sanyo Medicool, PHC Holdings Corporation, Tokyo, Japan) until testing could be performed

The first set of camel milk was sampled from desert-fed camels (DFCs) managed under a traditional pastoral system and was sampled from the Nafud Al-Thuwayrat area, a known fertile desert region located approximately 260 km northwest of the capital, Riyadh, Saudi Arabia. The second set of camel milk samples was collected from farm-fed camels (FFCs), maintained under intensive farm management, and were sampled from a private commercial facility, Al-Rassan Farms, located in Al-Asyah, Al-Qassim region. The sampling site was located within a radius of approximately 420 km from the Riyadh city area. Data collected included age of camels, parity, record types of feeds offered as basal and supplementary forages, types of concentrates used, sources of forages, and farming systems practiced.

### 2.2. Preparation of Milk Fat Globular Membrane (MFGM) Samples

Five samples (50 mL) of camel milk from both groups (desert-fed camels (DFCs) (*n* = 5) and farm-fed camels (FFCs) (*n* = 5)) were taken. The temperature of the samples was controlled at 2–8 °C during the procedures according to Saadaoui et al. and Pisanu et al. [31,32] with minor modifications. Samples were initially centrifuged at 4000 rpm (Eppendorf 5425 R, Eppendorf SE, Hamburg, Germany) for 35 min to separate the cream layer, which was then harvested into fresh 50 mL Falcon tubes. To facilitate fat melting and cluster separation, five volumes of 1M PBS (composed of 4.3 mM Na_2_HPO_4_, 2.7 mM KCl, 1.8 mM KH_2_PO_4_, and 137 mM NaCl, pH 7.4 (Sartorius PB-11, Sartorius AG, Göttingen, Germany)) were added. The mixture was incubated in a 37 °C water bath (Cole-Parmer, 625 East Bunker Court, Vernon Hills, IL, USA) for 20 min with periodic agitation. To eliminate residual casein and whey proteins, the samples underwent a wash cycle comprising two centrifugations with PBS and a final centrifugation with Milli-Q water (4000 rpm, 35 min each). The fat phase was transferred to a clean tube after every step. Following overnight storage at 2–8 °C (Sanyo Medicool, PHC Holdings Corporation, Tokyo, Japan), the samples were homogenized with sterile plastic beads via vortexing (60 Hz, 3 min). Finally, the samples were warmed to 37 °C for 10 min and centrifuged at 4000 rpm for 30 min. The supernatant oil layer was discarded, and the resulting white pellet containing the MFGM was weighed and frozen at −20 °C for subsequent analysis [31,32].

### 2.3. Extraction of MFGM Proteins

Using a modified method based on [33,34], MFGM pellets were lysed in a buffer containing 30 mM Tris, 7 M urea, 2 M thiourea, 2% CHAPS, and protease inhibitors with final pH 8.8 (Sartorius PB-11, Sartorius AG, Göttingen, Germany). Following a 1 h incubation with vortexing, samples were heated (95 °C, 5 min) and centrifuged (12,000 *g* for 15 min) (Eppendorf 5430 R, Eppendorf SE, Hamburg, Germany). The supernatant was treated with 50 mM DTT and further centrifuged at 20,000 *g* for 40 min at 4 °C. The resulting soluble protein fraction was purified using a 2D Clean-Up Kit (GE Healthcare, Uppsala, Sweden), and the pH was adjusted to 8.5 with 100 mM NaOH. Total protein concentration was determined using the 2D-Quant Kit (GE Healthcare, Chicago, IL, USA).

### 2.4. Sample Labeling with Cyanine Dyes

Fifty micrograms of protein from each MFGM-DFC (*n* = 5) and MFGM-FFC (*n* = 5) sample were labeled using the CyDye™ DIGE minimal labeling protocol (GE Healthcare, Chicago, IL, USA). Following a 30 minute incubation on ice in the dark, the reaction was terminated with 10 mM lysine (1 µL). Samples were randomized and labeled with either Cy3 or Cy5. Simultaneously, a pooled internal standard containing equal amounts of total protein from all samples was labeled with Cy2. The distribution of samples across the gels is provided in Table S1.

### 2.5. One- and Two-Dimensional Difference Electrophoresis (2D-DIGE)

One-dimensional separation was carried out using Immobiline Dry Strips (24 cm, pH 3–11; GE Healthcare, Uppsala, Sweden). The isoelectric focusing (IEF) strips were prepared for the second dimension through a two-step equilibration process. First, the strips were incubated for 15 min at room temperature (RT) with gentle stirring in a solution containing 5 mM Dithiothreitol (DTT), 6 M urea, 5 mM Tris–HCl (pH 8.8), 30% glycerol, and 2% SDS (65 mM). Subsequently, the strips were equilibrated for another 15 min in the identical buffer, but with the substitution of DTT for 250 mM Iodoacetamide (IAA). Second-dimension separation was performed using Sodium Dodecyl Sulfate Polyacrylamide Gel Electrophoresis (SDS-PAGE) on 12.5% fixed gels. This separation was conducted using an Ettan Dalt Six device (GE Healthcare, Uppsala, Sweden), following the methodology detailed by Alfadda et al. [35]. Further, the 2D-DIGE gels were scanned with a Sapphire Biomolecular Imager (Azure Biosystems, Dublin, OH, USA) and digitalized with the Sapphire Capture system image analysis software (Azure Biosystems, Dublin, OH, USA).

### 2.6. Statistical Analysis

The two-dimensional difference gel electrophoresis (2D DIGE), gel images were processed using Progenesis SameSpots software (Nonlinear Dynamics, Tyne, UK) to compare protein expression profiles between the DFC and FFC-MFGM samples. Initially an automated spot detection method was applied across all five gels. All detected spots were subsequently verified and manually confirmed. Normalized spot volumes were utilized for quantification to identify proteins that were differentially expressed between the groups. Proteins were deemed differentially expressed if the ratio of their normalized volumes between the groups met a ≥1.5-fold change cut-off. The statistical significance of the differences between the DFC and FFC-MFGM groups was determined and a threshold of *p* ≤ 0.05 was used to define statistical significance.

### 2.7. Protein Identification by MALDI-TOF MS

The Coomassie-stained gel spots were first washed and subjected to in-gel tryptic digestion to break the proteins into smaller peptide fragments, following established procedures [34,35]. A 1 µL aliquot of the resulting mixture of tryptic peptides from each protein was then applied to a MALDI target plate (384 Anchorchip MTP 800 µm Anchorchip; Bruker Daltonik, Bremen, Germany). MALDI-MS and tandem mass spectrometry (MS/MS) spectra were acquired using an ultrafleXtreme TOF mass spectrometer equipped with a LIFT-MS/MS device (Bruker Daltonics, Bremen, Germany). The instrument was operated with a reflector voltage of 21 kV and a detector voltage of 17 kV. Calibration of the peptide mass fingerprints (PMFs) was performed using a standard (peptide calibration standard II, Bruker Daltonics, Bremen, Germany) [33,36]. MS data processing was carried out using Flex Analysis software (version 2.4, Bruker Daltonics, Bremen, Germany), and interpreted with BioTools software (version 3.2, Bruker Daltonics, Bremen, Germany). The generated peptide masses were searched against a protein database using the Mascot search algorithm (version 2.0.04; Matrix Science Ltd., London, UK). Identified proteins were accepted as positive hits if they achieved a Mascot score exceeding 56 and met a statistical significance threshold of *p* < 0.05.

### 2.8. Bioinformatics Analysis

A Protein–Protein Interaction (PPI) network of the differentially expressed proteins was constructed using the STRING database (https://string-db.org/ accessed on 20 May 2023). This network was specifically used to analyze the interactions and functions of the milk fat globule membrane (MFGM) proteins identified as significantly different between the DFC and FFC-MFGM samples. The STRING database allows for the conversion of UniProt IDs into the Ingenuity Knowledge Base, which is the most comprehensive, manually curated resource available. This software facilitated the determination of the functions and pathways most strongly associated with the list of proteins generated via mass spectrometry (MS). It achieved this by overlaying the experimental expression data onto networks derived from published protein–protein interactions. For detailed functional annotation, the UniProt accession IDs obtained for the proteins were uploaded to the PANTHER (Protein Analysis Through Evolutionary Relationships) classification system (http://www.pantherdb.org accessed on 24 May 2023). PANTHER was utilized to classify the identified proteins into various functional categories. The results for these categories were exported and subsequently visualized using Microsoft Excel to create pie charts and bar graphs. These graphical representations illustrate the distribution of proteins across different categories, comparing the percentage of gene hits to the total functional hits obtained from the PANTHER classification data.

### 2.9. Hierarchical Clustering and Statistical Analysis

Proteins were grouped by their expression patterns using hierarchical clustering by a commercial Progenesis “SameSpots” software, version 3.3 (Nonlinear Dynamics, Newcastle upon Tyne, UK). This unsupervised, multivariate analysis method yields a true snapshot of protein activity across the experiment by organizing proteins based on their expression patterns. The output, an interactive dendrogram tree, visually represents the expression profiles for all significant proteins (both increased and decreased in abundance) across all samples, with proteins displaying similar expression profiles clustering closely together.

## 3. Results

### 3.1. Characteristics of Study Samples

The characteristics of the study samples (DFCs and FFCs) including age, types of feeds, types of concentrates used, sources of forages, and farming systems practiced are summarized in Table 1.

### 3.2. Quantitative Proteomics: Fluorescent Labeling and Differential Gel Electrophoresis

**Figure 1:**
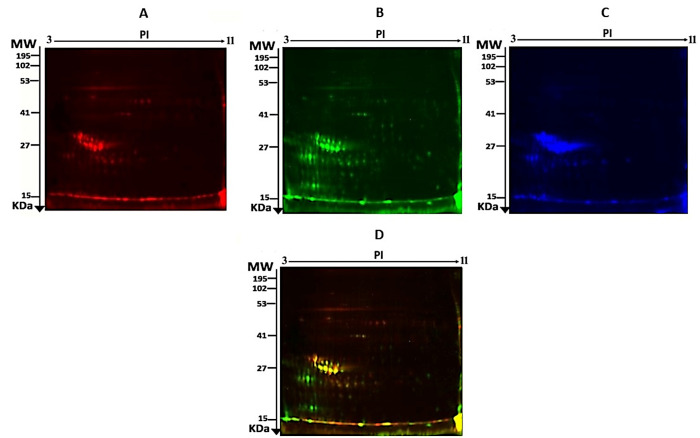
Representative two-dimensional difference gel electrophoresis (2D-DIGE) fluorescent protein profiles. The figure displays the fluorescent protein profiles of the milk fat globule membrane (MFGM) samples used for quantitative analysis: (**A**) MFGM from the desert-fed camels group labeled with Cy5 (red); (**B**) MFGM from the farm-fed camels group labeled with Cy3 (green); (**C**) the pooled internal control sample labeled with Cy2, used for cross-gel normalization; and (**D**) the merged image of the desert-fed camels/farm-fed camels samples (Cy5/Cy3), which allows for direct visual assessment of differential expression.

**Figure 2:**
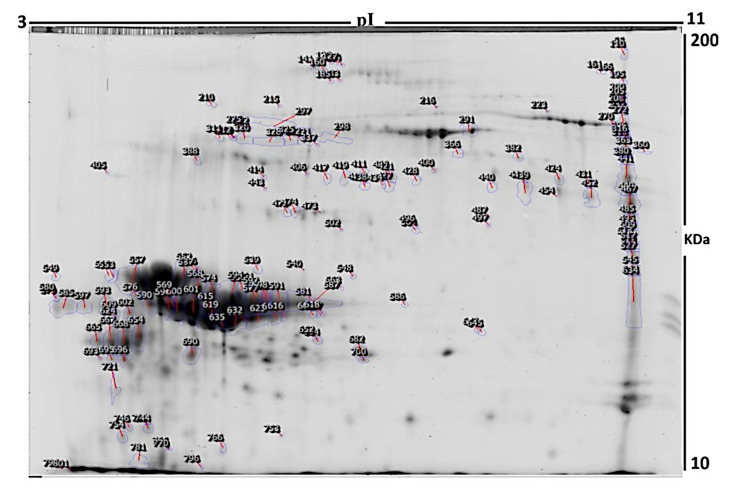
The two-dimensional difference gel electrophoresis analysis; the numbered spots on the figure correspond to proteins that show a differential abundance (a significant change in quantity) between the milk samples from desert-fed camels and farm-fed camels.

To evaluate protein expression differences in the milk fat globule membrane (MFGM) between the DFC and FFC groups, 2D-DIGE was performed using five biological replicates per group. Representative fluorescent profiles for the MFGM-DFC (Cy5-labeled) and MFGM-FFC (Cy3-labeled) samples are displayed in Figure 1A and Figure 1B, respectively. A pooled internal standard labeled with Cy2 (Figure 1C) was utilized to normalize data across all gels, ensuring robust quantitative analysis. Following the merging of Cy3 and Cy5 channels (Figure 1D), manual validation was conducted to eliminate artifacts. Of the 802 mapped protein spots, 120 exhibited significant differential expression (*p* ≤ 0.05; fold change ≥ 1.5) between the two groups (Figure 2). In the overlaid images, proteins from the DFC group appear red (Cy5), while those from the FFC group appear green (Cy3). Yellow spots indicate proteins with equivalent isoelectric points, molecular weights, and fluorescence intensities across both samples. The high reproducibility of spot patterns across all five replicates facilitated precise alignment and comparative analysis. Ultimately, the 120 significant spots were manually excised from preparative gels for identification via mass spectrometry.

### 3.3. Mass Spectrometry and Identification of Proteins

In total, 40 out of the 120 protein spots excised from the preparative gel and identified by peptide mass fingerprint (PMF). Mascot database searching against SWISS-PROT successfully identified 27 unique proteins from the MALDI-TOF MS data with high confidence scores (Table 1 and Table S2). Proteins identified by PMF exhibited sequence coverage between 10% and 44%. In several instances, variants of a single protein were identified at different positions across the gel (Table 2, Figure 2). Of the 40 proteins identified, 9 protein spots were upregulated and 31 were downregulated in desert-fed camel (Table 2, Figure 2). The significantly upregulated proteins included Alpha-S2-casein (2.37-fold, *p* = 0.003), Synaptonemal complex central element protein 1 (2.01-fold, *p* = 0.005), TSC22 domain family protein 3 (6.57-fold, *p* = 0.028), Nuclear pore complex protein Nup93 (4.43 -fold, *p* = 0.011), and Cytochrome c oxidase assembly protein COX16 homolog, mitochondrial (1.93-fold, *p* = 0.022). The significantly downregulated proteins included Alpha-S1-casein (−8.05-fold, *p* = 0.035), Lactotransferrin (−1.5-fold, *p* = 0.003), Lactadherin (−1.64-fold, *p* = 0.024), Casein kinase I isoform alpha (−1.5-fold, *p* = 0.012), and NADH dehydrogenase [ubiquinone] flavoprotein 2, mitochondrial (−1.5-fold, *p* = 0.029); a full list is provided in Table 2. Among the identified proteins, the presence of Actin, cytoplasmic 1, Alpha-S1-casein, Alpha-S2-casein, Lactotransferrin, and Synaptonemal complex central element protein 1 across various gel coordinates suggests the occurrence of proteolysis or post-translational modifications, resulting in multiple protein species (Table 2).

### 3.4. Principal Component Analysis

**Figure 3:**
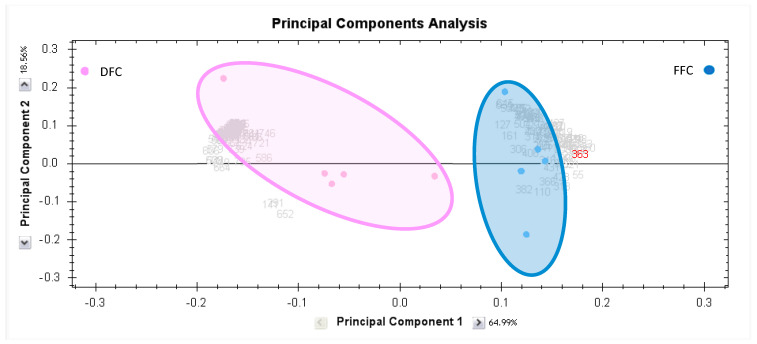
Principal Component Analysis of the proteomic dataset. Pink dots denote the milk samples from desert-fed camels (*n* = 5), and blue dots indicate the farm-fed camels milk samples (*n* = 5). Together, these explained 83.55% (PC1 64.99%; PC2 18.56%) of the selected spots’ variability values. Colored dots and numbers represent gels and spots, respectively.

Principal Component Analysis (PCA) was used to clearly visualize the differences between the desert-fed camel (DFC) and farm-fed camel (FFC) study groups, as depicted in the score plot of Figure 3, where samples are color-coded by group. The key finding from the PCA model is that the DFC (red circles) and FFC (blue circles) samples clustered distinctly, proving that their proteomics profiles are significantly different. This separation is largely driven by Principal Component 1 (PC1), which alone accounts for 64.99% of the variance in the data. Further supporting this difference, hierarchical clustering analysis revealed distinct clustering between the two groups based on their expression patterns. This approach additionally demonstrated that the feeding environment induces a systematic shift in the MFGM protein pattern rather than merely altering individual protein levels (Figure S1A,B).

### 3.5. Protein–Protein Interaction (PPI) Network Construction

**Figure 4:**
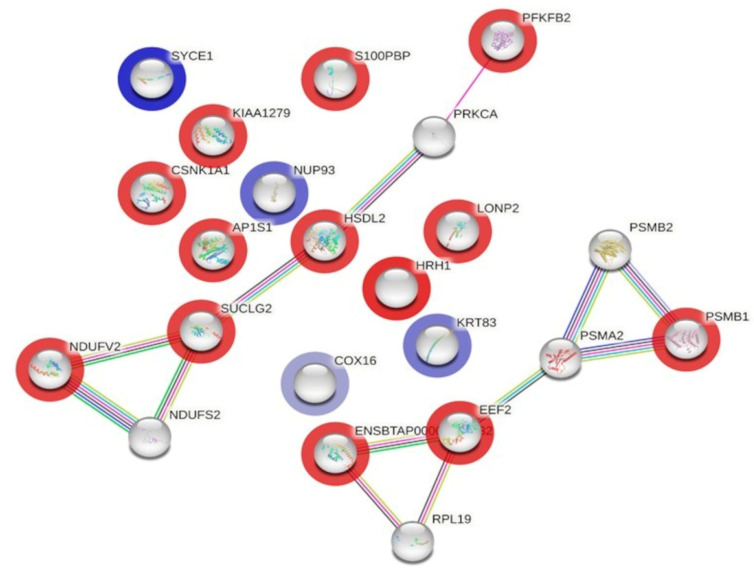
The Search Tool for the Retrieval of Interacting Genes/Proteins (STRING) interaction network shows the protein–protein interactions between the different identified proteins. The network allows the visualization of the relationship between differentially expressed milk proteins found in the desert-fed camel (DFC) and farm-fed camel (FFC) groups. The nodes (proteins) in the network are color-coded to indicate changes in expression: blue halo signifies upregulation; red halo indicates downregulation. Proteins without a halo are predicted by the STRING database as potential targets that functionally coordinate with the measured differentially expressed proteins. The edges (lines) connecting the nodes specify the type of evidence supporting the interaction: solid black indicates co-expression, green indicates gene neighborhood, dark blue denotes gene co-occurrence, and purple represents experimentally determined interactions.

The STRING protein–protein interaction database was used to identify known and predicted interactions among the differentially expressed proteins previously found through 2D-DIGE proteomics. The identified protein–protein interaction network was presented in Figure 4. We identified two networks; the network predicted that the interaction between hydroxysteroid dehydrogenase-like protein 2, NADH dehydrogenase [ubiquinone] flavoprotein 2, mitochondrial, and 6-phosphofructo-2-kinase/fructose-2,6-bisphosphatase 2 would be downregulated in DFC milk. The second network predicted that the interaction between elongation factor 2 and proteasome subunit beta type-1 would also be upregulated in DFC milk (Table S3).

**Figure 5:**
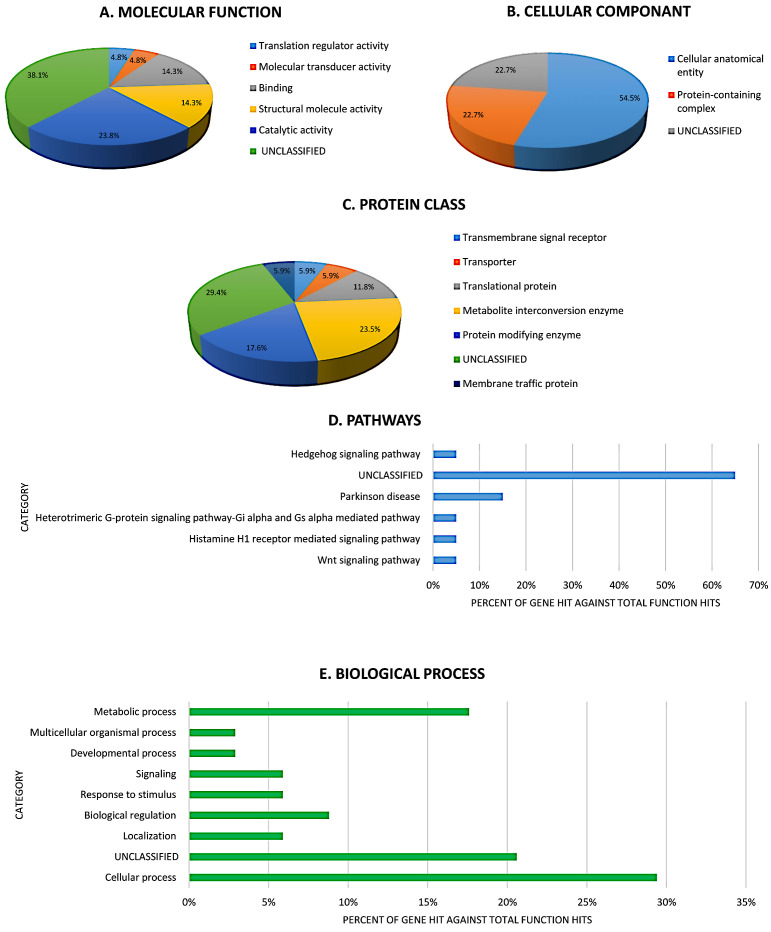
Percentage comparison of proteins identified in the milk fat globule membrane (MFGM) fraction of camel milk based on their function and location using the PANTHER (protein analysis through evolutionary relationships) classification system (http://www.pantherdb.org). (**A**) Molecular function, (**B**) cellular component, (**C**) protein class, (**D**) pathways, and (**E**) biological process.

The protein analysis through an evolutionary relationships (PANTHER) system was used for the classification of identified proteins according to their molecular functions (Figure 5A), cellular components (Figure 5B), protein class (Figure 5C), pathways (Figure 5D), and biological processes (Figure 5E). The functional category showed that most of the differentially expressed proteins identified were unclassified (38.10%) followed by catalytic activity (23.8%) (Figure 5A). The majority of the identified proteins were located in the cellular anatomical entity (54.5%) (Figure 5B). In protein class, the majority of the identified proteins were metabolite interconversion enzyme (23.5%) and protein-modifying enzyme (17.6%) (Figure 5C). The identified proteins were included in Wnt signaling pathways, Hedgehog signaling pathways, etc. (Figure 5D; Table S3). With regard to biological processes, the majority of the identified proteins were involved in cellular processes (29.4%) followed by metabolic processes (17.6%) and biological regulation (8.8%) (Figure 5E).

## 4. Discussion

Camel milk stands out for its complex and adaptive nutritional profile, shaped by camel physiology and the environments in which camels often live. There is limited information related to changes in milk composition based on these changes. Our present study provides the first comprehensive analysis of differentially expressed milk MFGM proteins in *Camelus dromedarius*, Waddah breed, between two different feeding practices, DFCs and FFCs. 2D-DIGE mass spectrometric analysis revealed significant differences in the composition of the proteins in the MFGM fraction. A large number of the identified proteins were observed to be predominantly down regulated in the camels maintained under desert feeding compared to FFC. These included those involved in regulating immune response, protein homeostasis, and stress response cell viability. Lactotransferrin, Lactadherin, TLR2, αS1-casein, and mitochondrial/antioxidant proteins (NDUFV2, PDH-E1α, SUCLG2, SOD1) were reduced alongside translational and proteasomal markers (EF2, RPL13a, proteasome β1) and cytoskeletal/trafficking elements (actin, AP-1 complex σ1A, CK1-α) in DFC compared to FFC. In contrast, αS2-casein, COX16, TSC22D3, Nup93, EPO, and keratin type 2 were higher in DFC compared to FFC. The observed differences in MFGM proteomic profiles between the two groups may indicate underlying cellular-level differences related to feeding environments.

Camel milk is known to contain bioactive proteins and peptides (e.g., lactoferrin, immunoglobulins) that were observed to modulate immune responses and inhibit pathogen growth [27,28]. The MFGM layer surrounding the milk fat globules is enriched in lipids, peptides, and proteins such as butyrophilin, xanthine oxidoreductase, Lactadherin, mucins, and Lactotransferrin. These proteins are known to play an active role in diverse metabolic functions, including fat secretion, immune protection, antimicrobial activity, and antioxidant defense [37,38].

We observed differential regulation of α-caseins between the two groups, with αS1-casein being downregulated and αS2-casein upregulated in camels under desert-based feeding (DFC) compared with farm-based feeding (FFC). Overall, camel milk is known to contain lower levels of caseins than cow’s milk, which is associated with improved digestibility and reduced allergenicity. Casein is a major phosphoprotein that provides essential amino acids, binds and transports calcium and phosphorus for bone health, and promotes satiety due to its slow digestion. It represents the most abundant protein fraction in milk, accounting for approximately 80% of total milk protein. Variations in casein concentration can influence casein micelle size and affect both the quantity and quality of milk fatty acids. Additionally, different casein types vary in structure, mineral-binding capacity, and digestibility. At the cellular level alpha-S1-casein mediates the ER-to-Golgi export of the casein fraction. It also drives the formation of casein micelles and serves as a critical vehicle for calcium phosphate delivery in milk [39]. Casein content in the milk, along with calcium, determines the coagulation properties of milk and curd firmness. A decrease in the levels of αS1-casein could account for the decrease in camel milk curd firmness and calcium content, while an increase in αS2-casein might improve heat stability and calcium solubility. The lower αS1-casein content in DFC may contribute to its diluted protein profile, which is not beneficial for cheese making, but on the other hand favors easier digestibility. It was observed in bovine milk that higher α-S1-casein content correlated with larger, more hydrated casein micelles. Similar diet-dependent variations in casein profiles have been reported in cows and goats, where higher forage intake or environmental stress influence total protein content and casein heterogeneity. Milk from goats grazing on cultivated pasture was found to have significantly higher alpha-S1-casein content compared to the milk from those that were fed on the rangelands [40,41]. The altered αS1/αS2 ratio therefore reflects nutritional modulation of milk composition by feeding system.

We also identified a decrease in abundance in the immune-related proteins such as Lactotransferrin, Lactadherin (also known as MFG-E8), Toll-like receptor 2 (TLR2), and the histamine H1 receptor in the MFGM of DFCs compared to FFCs. These proteins play central roles in the milk’s natural immune system. Lactotransferrin is a multifunctional glycoprotein abundant in camel milk, known for its iron-binding capacity, which limits bacterial growth by sequestering free iron. It exerts antibacterial, antiviral, antioxidant, and immunomodulatory effects, playing a crucial role in protecting from infection and inflammation. Its consistent downregulation in DFCs suggests a lower level of innate immune activation in the mammary gland between DFCs and FFCs. Lactadherin, also known as milk fat globule-EGF factor 8 proteins, is a multifaceted glycoprotein present in milk. Its diverse biological functions encompass roles in immunity, cell adhesion, angiogenesis, and stimulation of the immune system. It also helps in maintenance of intestinal epithelial homeostasis and the promotion of mucosal healing link to apoptotic cells so they can be recognized by phagocytes for engulfment. This protein supports intestinal epithelial health, facilitates clearance of apoptotic cells, and has antiviral properties. Milk fat globule size is an important predictor of protein signatures, surface polarity, and fatty acid and phospholipid composition. The decrease in Lactadherin indicates smaller milk globule size in comparison to the FFC [42,43]. Toll-like receptor 2 (TLR2), a host defense protein which recognizes bacterial lipoproteins and triggers immune signaling, was also reduced. TLR2 presence in milk has been associated with enhanced immune protection. The proteins identified in this study align with those previously characterized in human and bovine milk; they are recognized for their capacity to sequester bacterial lipopolysaccharides (LPSs) and exhibit significant antimicrobial properties [28,44]. A similar trend was seen for the histamine H1 receptor, involved in inflammatory signaling and allergic response regulation. Their downregulation suggests that the mammary immune environment of desert-fed camels is less stimulated than FFCs. The observed pattern implies that milk from FFCs carries a stronger immune and antimicrobial signature, enhancing its biological protection and possibly contributing to the therapeutic reputation of traditional camel milk.

Proteins involved in mitochondrial energy metabolism including NADH dehydrogenase [ubiquinone] flavoprotein 2 (NDUFV2), pyruvate dehydrogenase E1 α, and succinate-CoA ligase β-subunit (SUCLG2) were significantly downregulated in the DFC compared to FFCs. These enzymes are key components of the tricarboxylic acid cycle and the oxidative phosphorylation pathway, which generate cellular energy; ATP [45]. Their reduced abundance suggests that mammary epithelial cells in DFCs operate at a lower oxidative metabolic rate compared to FFCs that have constant nutrient supply, lower physical activity, and reduced environmental stress. Consequently, their milk may incorporate higher levels of metabolic enzymes, reflecting enhanced mitochondrial activity within the mammary gland. In contrast, desert-grazing camels are exposed to varying temperatures, prolonged periods of walking, and seasonal feed fluctuations. These conditions, all of which increase energy expenditure and stimulate oxidative metabolism, probably lead to increased utilization of these proteins. Similarly, the antioxidant enzyme superoxide dismutase (SOD1) was lower in DFCs compared to FFCs. This enzyme neutralizes reactive oxygen species and protects cellular membranes including the MFGM from oxidative damage. The decline in SOD1 under desert feeding indicates lower oxidative stress and also reduced antioxidant content in milk, potentially affecting its oxidative stability and bioactive potential [10]. Moreover, levels of hydroxysteroid dehydrogenase-like protein 2, which participates in steroid metabolism, were lower in DFCs compared to FFCs, implying reduced hormone conversion activity under desert feeding. Together, these patterns suggest that intensive systems favor cellular maintenance and hormonal regulation, while extensive grazing enhances stress-related metabolic responses. The decrease in abundance of key metabolic and antioxidant proteins, including enzymes that combat oxidative damage such as superoxide dismutase, Lactotransferrin, and NADH dehydrogenase within the MFGM fraction in DFC, can be attributed to the nutritional and oxidative stress they experience. This adaptive metabolic suppression means fewer resources are invested in growth and rapid metabolism, and more energy is conserved for cellular maintenance and survival during periods of environmental harshness.

Several proteins associated with translation and proteolysis, such as elongation factor 2 (EF2), 60S ribosomal protein L13a, and the proteasome subunit β type-1, were also downregulated under desert feeding. This cluster of proteins reflects cellular activity and protein turnover, in our case probably related to the mammary gland. The combined increase in these three components indicates an increase in the cell’s ability to produce new proteins (via decreased EF2 and L13a involvement in translation) and degrade old/damaged ones (via decreased PSMB1) as a part of protein homeostasis. Their reduced abundance in DFCs suggests that mammary cells of desert-fed camels are in a more stable and less active physiological state compared to FFC. Cytoskeletal and membrane-trafficking proteins, including actin (cytoplasmic 1), AP-1 complex subunit sigma-1A, and casein kinase I isoform α, were also downregulated under desert feeding. Actin and AP-1 are essential for vesicle movement and secretion of milk fat globules, while CK1 regulates multiple signaling and transport processes. Their reduced expression points to higher vesicular transport activity and increased membrane remodeling in FFCs in comparison to DFCs.

Although desert-fed camels showed an overall reduction in many immune, antioxidant, and metabolic proteins, several proteins were specifically elevated under desert grazing, and their functions provide important insight into the unique physiological pressures faced by these animals. The upregulation of TSC22D3 (GILZ) in desert-fed camels is particularly notable. GILZ is a glucocorticoid-responsive protein that rises during chronic environmental stress, dehydration, or prolonged activation of the hypothalamic–pituitary–adrenal axis. It is known to have strong anti-inflammatory and anti-apoptotic roles. Its increased expression indicates that desert feeding conditions elicit sustained endocrine modulation, allowing the mammary gland to restrain inflammation and cellular damage while conserving metabolic energy. A similar adaptive role is suggested by the increase in Nup93, a nuclear pore complex protein involved in maintaining nuclear membrane integrity and regulating gene transport during cellular stress. Elevated Nup93 in DFCs implies that the mammary epithelium is engaged in stabilizing nuclear–cytoplasmic communication to preserve essential functions in the face of heat, probable nutrient fluctuations, and/or stress adaptation such as in the desert environment.

The rise in keratin isoforms, which are structural proteins of epithelial cells, also reflects a protective response to the desert physical environment. Desert conditions often cause microdamage and accelerated turnover of epithelial tissues; therefore, keratin upregulation suggests active reinforcement of the mammary epithelium to maintain barrier integrity and sustain milk secretion. The increased presence of erythropoietin (EPO) also aligns with this physiological context. EPO is typically induced under hypoxic or dehydration-related stress, where it promotes cellular survival and reduces apoptosis. Its elevation in desert-fed camels indicates that the mammary gland relies on EPO-mediated cytoprotection to cope with the combined pressures of heat stress, lower water intake, and fluctuating forage availability.

Another protein elevated in desert-fed camels was cytochrome c oxidase assembly factor COX16, which supports the organization of Complex IV in the mitochondrial electron transport chain. This selective increase may represent a compensatory mechanism to maintain minimal mitochondrial efficiency when broader metabolic pathways are downregulated. Similarly, the increase in αS2-casein, compared with the decrease in αS1-casein, suggests a shift toward a casein profile optimized for mineral binding and thermal stability qualities advantageous in arid conditions. Together, the proteins elevated in desert-fed camels reveal a coherent adaptive pattern. Instead of investing energy into producing abundant immune or metabolic proteins, the mammary gland enhances regulatory, structural, and cytoprotective pathways that help maintain epithelial integrity and safeguard essential cellular processes during chronic environmental stress.

From a human nutrition perspective, the MFGM plays a central role in defining the bioactive and health-promoting properties of camel milk beyond its basic nutrient content. The feeding-system-dependent differences observed in this study indicate that milk from FFCs is enriched in immune-related and antioxidant proteins, such as Lactotransferrin and Lactadherin, which may support antimicrobial defense, oxidative balance, and intestinal health. On the other hand DFCs exhibited an MFGM profile characterized by reduced metabolic and immune proteins but increased regulatory, structural, and cytoprotective proteins, including an altered casein profile [46].

While the results of this study provide informative preliminary observations, they should be viewed within the context of our exploratory pilot, proof-of-concept design. In particular, the present work focused on proteomic characterization and did not include direct physiological or endocrine measurements of stress or hydration markers. Consequently, interpretations linking specific MFGM proteins to environmental or metabolic adaptation are based on their established functional roles rather than direct physiological evidence in the sampled animals. From a consumer and dairy industry perspective, these findings suggest that feeding systems may influence the functional and nutritional profile of camel milk. Milk from FFCs, with higher levels of immune and antioxidant-related MFGM proteins, may be more suitable for functional foods, therapeutic nutrition, or products targeting immune and gut health. In contrast, milk from DFCs, characterized by an altered casein composition and reduced αS1-casein, may favor improved digestibility and lower allergenic potential, making it potentially attractive for direct consumption and sensitive populations. Collectively, these observations indicate that feeding practices could be explored as a strategy to tailor camel milk for specific consumer markets and value-added dairy applications. Future studies incorporating larger, well-characterized camel cohorts alongside integrated physiological, metabolic, and endocrine measurements will be valuable to further substantiate and refine these observations. Despite this, the current study serves as an important proof of concept demonstrating the sensitivity of the MFGM proteome to feeding environment.

## 5. Conclusions

This study provides the first comparative proteomic analysis of the MFGM of *Camelus dromedarius* (Waddah breed) under desert-fed and farm-fed systems in Saudi Arabia. Farm feeding favored enrichment of immune-, antioxidant-, and metabolism-associated proteins, whereas desert feeding promoted regulatory and cytoprotective signatures consistent with environmental adaptation. These findings demonstrate that distinct feeding practices markedly alter the MFGM proteome composition and functional quality of camel milk. Overall, the findings highlight that feeding and management practices modulate the molecular quality of camel milk, and that optimizing production systems to preserve both biofunctional richness and yield is essential for sustaining the nutritional and economic value of the emerging camel dairy sector. Overall, the data indicate that desert-grazing camels produce milk with a richer array of immune, metabolic, and antioxidant proteins, reflecting high adaptive activity and environmental stress exposure.

## Figures and Tables

**Table 1:** The characteristics of the study samples.

	Desert-Fed Camels (DFCs) *Camelus dromedaries* Species (*n* = 5)	Farm-Fed Camels (FFCs) *Camelus dromedaries* Species (*n* = 5)
Breed	Waddah	Waddah
Age (years) (mean ± SD)	3.8 ± 0.74	4.2 ± 0.74
Parity (years)	1–2	1–2
Types of feed	sparse grasses, shrubs, and thorny bushes (like halophytes or salt-tolerant plants) e.g.: *Cenchrus ciliaris* *Cressa cretica* *Heliotropium bacciferum* *Tribulus* *Savignya Parviflora*	cultivated fodder such as Bermuda hay, Alfalfa, crop residues like cluster bean, wheat, groundnut straw, barley, wheat bran, yellow corn, soybean powder, locally cultivated fruits and vegetables, salt blocks, vitamin and mineral supplements.
Water sources	brackish water, temporary wells, water holes	troughs, taps, boreholes

Age is presented as mean ± standard deviation. DFCs, desert-fed camels; FFCs, farm-fed camels; SD; standard deviation.

**Table 2:** Differentially abundant milk fat globule membrane proteins identified between desert-fed and farm-fed camels by 2D-DIGE and MALDI-TOF. Average protein ratios and fold changes were determined using 2D-DIGE (*p* < 0.05). Proteins were identified by MALDI-TOF MS against the SwissProt database (Taxonomy: Other Mammalian).

Sl No:	Spot No ^a^	Accession No	Protein Name	MASCOT ID	*p* Value ^b^	Ratio ^c^ DFCs/FFCs	Exp ^d^
1	439	Q3SX23	**Lon protease homolog 2, peroxisomal**	LONP2_BOVIN	0.017	−1.5	DOWN
2	502	P84336	**Actin, cytoplasmic 1**	ACTB_CAMDR	0.005	−1.5	DOWN
3	467	P04394	**NADH dehydrogenase [ubiquinone] flavoprotein 2, mitochondrial**	NDUV2_BOVIN	0.029	−1.5	DOWN
4	414	P84336	**Actin, cytoplasmic 1**	ACTB_CAMDR	0.048	−1.52	DOWN
5	561	P80220	**TSC22 domain family protein 3**	T22D3_PIG	0.028	6.57	UP
6	208	Q9TUM0	**Lactotransferrin**	TRFL_CAMDR	0.009	−1.5	DOWN
7	485	Q9TUM0	**Lactotransferrin**	TRFL_CAMDR	0.024	−1.5	DOWN
8	540	O97943	**Alpha-S1-casein**	CASA1_CAMDR	0.002	−1.5	DOWN
9	549	A5PJZ5	**Nuclear pore complex protein Nup93**	NUP93_BOVIN	0.011	4.43	UP
10	312	O97943	**Alpha-S1-casein**	CASA1_CAMDR	0.014	−1.66	DOWN
11	539	P26285	**6-phosphofructo-2-kinase/fructose-2,6-bisphosphatase 2**	F262_BOVIN	0.048	−1.5	DOWN
12	474	O97943	**Alpha-S1-casein**	CASA1_CAMDR	0.022	−1.5	DOWN
13	563	Q2TBX6	**Proteasome subunit beta type-1**	PSB1_BOVIN	0.004	−1.5	DOWN
14	311	O97943	**Alpha-S1-casein**	CASA1_CAMDR	0.031	−1.5	DOWN
15	135	P52900	**Pyruvate dehydrogenase E1 component subunit alpha, mitochondrial**	ODPA_SMIMA	0.024	−1.5	DOWN
16	388	O97943	**Alpha-S1-casein**	CASA1_CAMDR	0.034	−1.5	DOWN
17	553	O97943	**Alpha-S1-casein**	CASA1_CAMDR	0.035	−8.05	DOWN
18	548	Q3MHH3	**S100P-binding protein**	S1PBP_BOVIN	0.009	−1.5	DOWN
19	664	O97944	**Alpha-S2-casein**	CASA2_CAMDR	0.003	2.37	UP
20	652	O97944	**Alpha-S2-casein**	CASA2_CAMDR	0.007	3.85	UP
21	55	Q1JQ98	**AP-1 complex subunit sigma-1A**	AP1S1_BOVIN	0.036	−1.5	DOWN
22	282	P67827	**Casein kinase I isoform alpha**	KC1A_BOVIN	0.012	−1.5	DOWN
23	141	Q2NKS2	**Cytochrome c oxidase assembly protein COX16 homolog, mitochondrial**	COX16_BOVIN	0.022	1.93	UP
24	200	Q3SYU2	**Elongation factor 2**	EF2_BOVIN	0.019	−1.56	DOWN
25	585	A3FFS8	**Erythropoietin**	EPO_BOSMU	0.050	7.09	UP
26	306	P30546	**Histamine H1 receptor**	HRH1_BOVIN	0.02	−1.72	DOWN
27	216	A4FUZ6	**Hydroxysteroid dehydrogenase-like protein 2**	HSDL2_BOVIN	0.007	−1.5	DOWN
28	693	A4FUZ0	**Keratin, type II cuticular Hb3**	KRT83_BOVIN	0.011	3.76	UP
29	319	Q3SYS9	**KIF-binding protein**	KBP_BOVIN	0.004	−1.5	DOWN
30	363	Q9TUM0	**Lactotransferrin**	TRFL_CAMDR	<0.001	−1.54	DOWN
31	217	Q9TUM0	**Lactotransferrin**	TRFL_CAMDR	0.003	−1.5	DOWN
32	272	Q9TUM0	**Lactotransferrin**	TRFL_CAMDR	0.005	−1.5	DOWN
33	360	Q3SZ90	**60S ribosomal protein L13a**	RL13A_BOVIN	<0.001	−1.5	DOWN
34	232	Q9TUM0	**Lactotransferrin**	TRFL_CAMDR	0.007	−1.5	DOWN
35	325	Q3MHX5	**Succinate--CoA ligase [GDP-forming] subunit beta, mitochondrial**	UCB2_BOVIN	0.019	−1.5	DOWN
36	316	P79385	**Lactadherin**	MFGM_PIG	0.024	−1.64	DOWN
37	421	P00443	**Superoxide dismutase [Cu-Zn]**	SODC_HORSE	0.025	−1.5	DOWN
38	183	Q32LK9	**Synaptonemal complex central element protein 1**	SYCE1_BOVIN	0.005	2.01	UP
39	746	Q32LK9	**Synaptonemal complex central element protein 1**	SYCE1_BOVIN	0.050	6.73	UP
40	497	Q6T752	**Toll-like receptor 2**	TLR2_HORSE	0.015	−1.5	DOWN

DFCs, desert-fed camels; FFCs, farm-fed camels; ^a^ Protein accession number for SWISSPROT Database. ^b^ *p*-Value (ANOVA). ^c^ Ratio between the groups. ^d^ Protein expression between the groups.

## Data Availability

The original contributions presented in this study are included in the article/Supplementary Material. Further inquiries can be directed to the corresponding author.

## Supplementary Materials

The following supporting information can be downloaded at: https://www.mdpi.com/article/10.3390/foods15030506/s1. Table S1: Summary of experimental design. Table S2: Comprehensive list of significantly differentially abundant proteins identified via mass spectrometry. Table S3: Canonical pathways identified through STRING and PANTHER database analyses. Figure S1: The results of a hierarchical cluster analysis, for MFGM (milk fat globule membrane) proteins based on their expression profiles between the desert-fed camels (DFCs) group and the farm-fed camels (FFCs) group. Each line within the figure represents the standardized abundance of a single protein spot across all experimental gels. The analysis separated the proteins into two main clusters: (A) Cluster highlights 9 protein spots that show an increased abundance (upregulation) in the DFC group compared to FFC. (B) Cluster shows 31 protein spots that display a decreased abundance (downregulation), as determined by the Progenesis SameSpots software.
