# Supplementary material for: Proteomic Analysis Comparing Effect of Feeding Practices on the Milk Fat Globule Membrane Proteins from Camelus dromedarius
Source: Foods. 2026 Feb 1;15(3):506. doi: 10.3390/foods15030506 (PMC12897367; doi:10.3390/foods15030506)
Supplement: Supplementary file 1 [file foods-15-00506-s001.zip › foods-4081301-supplementary figure 1.pdf]

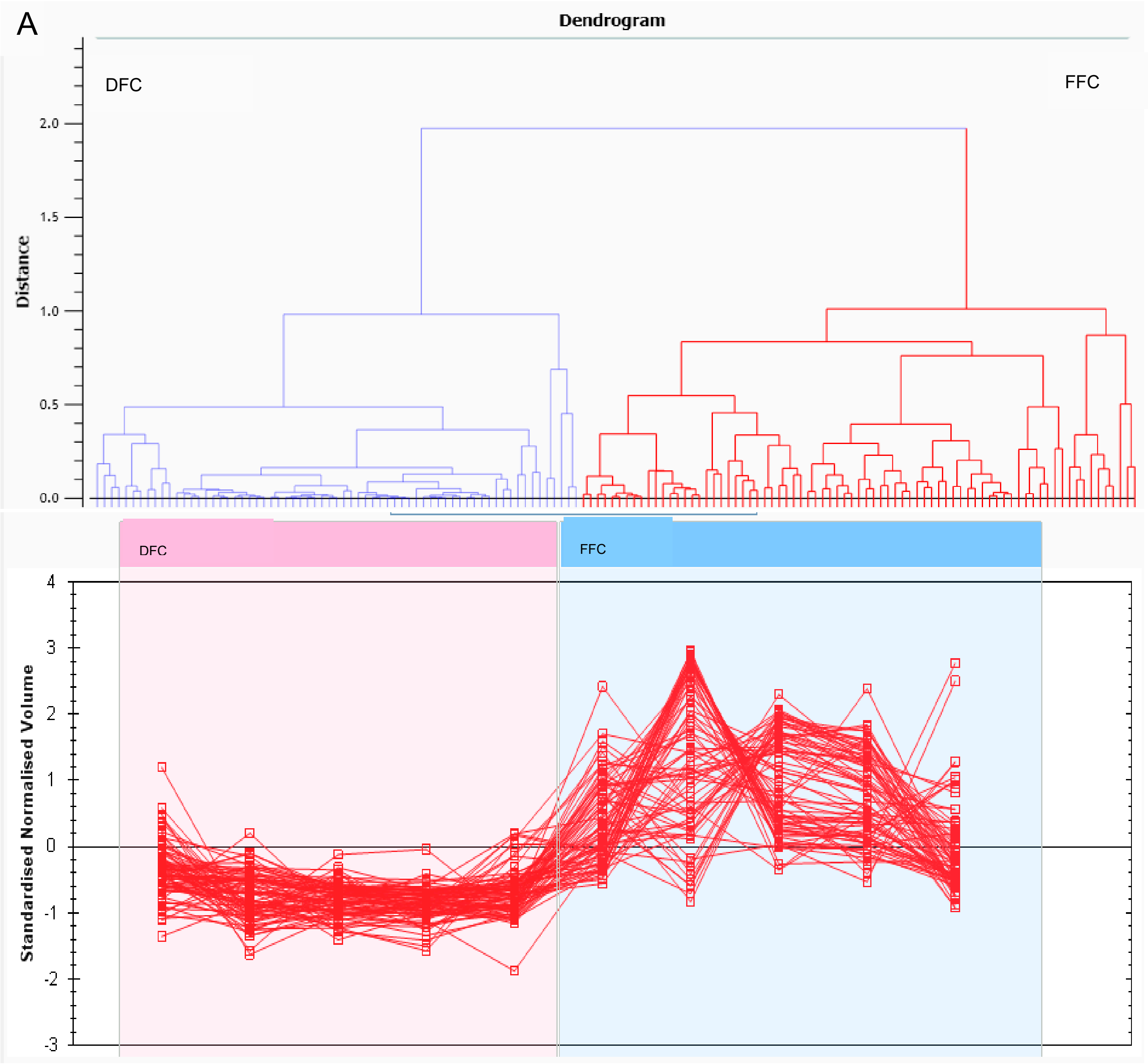

A
Dendrogram
Distance
2.0
1.5
1.0
0.5
0.0
DFC
FFC
DFC
FFC
Standardised Normalised Volume
4
3
2
1
0
-1
-2
-3

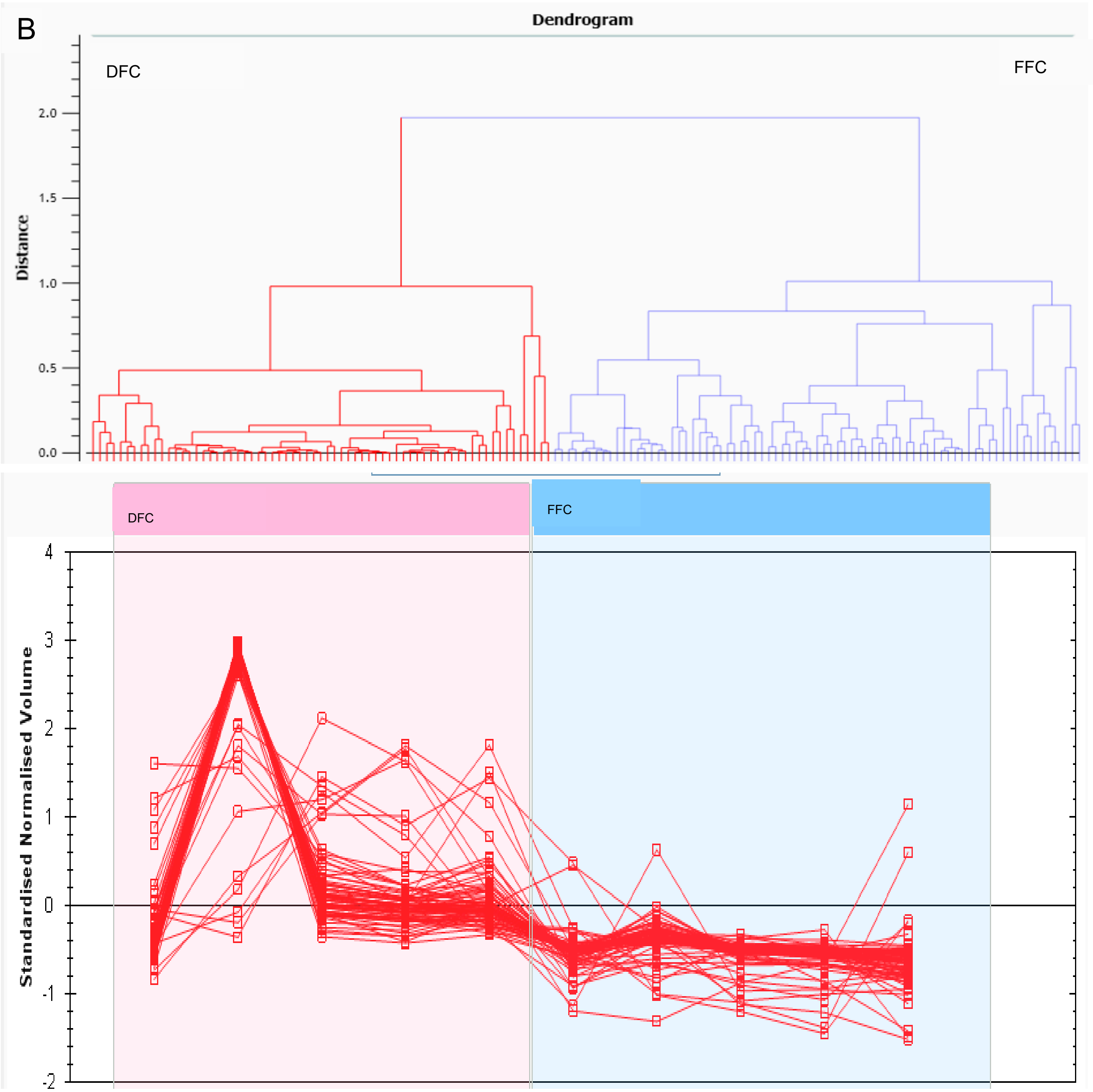

Figure S1: The results of a hierarchical cluster analysis, for MFGM (milk fat globule membrane) proteins based on their expression profiles between the desert fed camels (DFC) group and the farm fed camels (FFC) group
