# Supplementary material for: Proteomic Analysis Comparing Effect of Feeding Practices on the Milk Fat Globule Membrane Proteins from Camelus dromedarius
Source: Foods. 2026 Feb 1;15(3):506. doi: 10.3390/foods15030506 (PMC12897367; doi:10.3390/foods15030506)
Supplement: Supplementary file 1 [file foods-15-00506-s001.zip › foods-4081301-supplementary Tables S1 and S2.pdf]

Table S1. The distribution of samples in each gel

Gel	Cy3	Cy5	Cy2
Gel1	DFC1	FFC1	Pooled sample
Gel2	FFC2	DFC2	Pooled sample
Gel3	DFC3	FFC3	Pooled sample
Gel4	FFC4	DFC4	Pooled sample
Gel5	DFC5	FFC5	Pooled sample

DFC, desert fed camels; FFC, farm fed camels; Cy3, cyanine 3 (green laser channel) fluorophore-labeled; Cy5, cyanine 5 (red laser channel) fluorophore-labeled; Cy2, cyanine 2 (blue laser channel) fluorophore-labeled

Table S2. Mass spectrometry list of significant differentially abundant proteins

Sl no:	Spot No[a]	Accession No[b]	Protein Name	MASCOT ID	Pi[c]	MW[d]	Cov%	Score[e]
1	217	Q9TUM0	Lactotransferrin	TRFL_CAMDR	8.66	79158	10	52
2	363	Q9TUM0	Lactotransferrin	TRFL_CAMDR	8.66	79958	21	97
3	319	Q3SYS9	KIF-binding protein	KBP_BOVIN	5.36	72565	14	52
4	540	O97943	Alpha-S1-casein	CASA1_CAMDR	4.96	26902	43	98
5	502	P84336	Actin, cytoplasmic 1	ACTB_CAMDR	5.30	42174	29	67
6	664	O97944	Alpha-S2-casein	CASA2_CAMDR	6.00	23121	44	53
7	563	Q2TBX6	Proteasome subunit beta type-1	PSB1_BOVIN	8.26	26457	37	55
8	272	Q9TUM0	Lactotransferrin	TRFL_CAMDR	8.66	79158	44	171
9	183	Q32LK9	Synaptonemal complex central element protein 1	SYCE1_BOVIN	5.52	36981	25	53
10	652	O97944	Alpha-S2-casein	CASA2_CAMDR	6.00	23121	33	62
11	232	Q9TUM0	Lactotransferrin	TRFL_CAMDR	8.66	79158	29	114
12	208	Q9TUM0	Lactotransferrin	TRFL_CAMDR	8.66	79158	24	79

13	**549**	A5PJZ5	Nuclear pore complex protein Nup93	NUP93_BOVIN	5.50	93913	18	49
14	**693**	A4FUZ0	Keratin, type II cuticular Hb3	KRT83_BOVIN	5.43	55665	34	56
15	**282**	P67827	Casein kinase I isoform alpha	KC1A_BOVIN	9.50	37771	33	54
16	**325**	Q3MHX5	Succinate--CoA ligase [GDP-forming] subunit beta, mitochondrial	UCB2_BOVIN	7.51	47004	17	46
17	**312**	O97943	Alpha-S1-casein	CASA1_CAMDR	4.96	26902	27	57
18	**497**	Q6T752	Toll-like receptor 2	TLR2_HORSE	6.40	91190	29	48
19	**306**	P30546	Histamine H1 receptor	HRH1_BOVIN	9.15	56662	19	54
20	**439**	Q3SX23	Lon protease homolog 2, peroxisomal	LONP2_BOVIN	6.91	94764	19	56
21	**200**	Q3SYU2	Elongation factor 2	EF2_BOVIN	6.41	96276	30	80
22	**485**	Q9TUM0	Lactotransferrin	TRFL_CAMDR	8.66	79158	28	70
23	**561**	P80220	TSC22 domain family protein 3	T22D3_PIG	4.65	8707	41	47
24	**316**	P79385	Lactadherin	MFGM_PIG	6.15	46722	27	59
25	**467**	P04394	NADH dehydrogenase [ubiquinone] flavoprotein 2, mitochondrial	NDUV2_BOVIN	8.21	27575	44	53
26	**474**	O97943	Alpha-S1-casein	CASA1_CAMDR	4.96	26902	24	64
27	**141**	Q2NKS2	Cytochrome c oxidase assembly protein COX16 homolog, mitochondrial	COX16_BOVIN	9.73	12510	28	52
28	**553**	O97943	Alpha-S1-casein	CASA1_CAMDR	4.96	26902	30	121
29	**135**	P52900	Pyruvate dehydrogenase E1 component subunit alpha, mitochondrial	ODPA_SMIMA	6.51	41335	24	64
30	**421**	P00443	Superoxide dismutase [Cu-Zn]	SODC_HORSE	6.03	16232	20	51
31	**414**	P84336	Actin, cytoplasmic 1	ACTB_CAMDR	5.30	42174	42	83

**32**	**746**	Q32LK9	Synaptonemal complex central element protein 1	SYCE1_BOVIN	5.52	36981	27	60
**33**	**585**	A3FFS8	Erythropoietin	EPO_BOSMU	8.58	21419	36	50
**34**	**216**	A4FUZ6	Hydroxysteroid dehydrogenase-like protein 2	HSDL2_BOVIN	8.46	45519	27	49
**35**	**548**	Q3MHH3	S100P-binding protein	S1PBP_BOVIN	5.25	47375	18	46
**36**	**311**	O97943	Alpha-S1-casein	CASA1_CAMDR	4.96	26902	30	64
**37**	**388**	O97943	Alpha-S1-casein	CASA1_CAMDR	4.96	26902	37	146
**38**	**539**	P26285	6-phosphofructo-2-kinase/fructose-2,6-bisphosphatase 2	F262_BOVIN	8.50	61286	24	49
**39**	**360**	Q3SZ90	60S ribosomal protein L13a	RL13A_BOVIN	11.59	24391	26	49
40	55	Q1JQ98	AP-1 complex subunit sigma-1A	AP1S1_BOVIN	5.60	18818	29	64

[a]Protein accession number for SWISSPROT Database, [b]Theoretical isoelectric point, [c]Theoretical relative mass, [d] MASCOT coverage, [e] MASCOT score
